# Advantages of single-site laparoscopic orchiopexy for palpable undescended testes in children: a prospective comparison study

**DOI:** 10.1007/s00383-023-05630-8

**Published:** 2024-01-12

**Authors:** Zhilin Yang, Yingying He, Pengyu Chen, Tiejun Zhang, Zhicong Ke, Fenghao Sun, Guanglun Zhou, Weiguang Zhao, Shoulin Li

**Affiliations:** 1 Department of Urology and Laboratory of Pelvic Floor Muscle Function, Shenzhen Children’s Hospital, Futian District, Shenzhen, Guangdong 518000 P.R. China; 2 Department of Urology, Shenzhen Children’s Hospital, China Medical University, Shenzhen, Guangdong 518000 P.R. China

**Keywords:** Laparoscopic, Orchiopexy, Palpable, Single site, Undescended testis

## Abstract

**Purpose:**

To evaluate the feasibility of single-site laparoscopic orchiopexy for palpable undescended testes in children.

**Methods:**

We prospectively studied patients with undescended testes between July 2021 and June 2022. In total, 223 patients were included in our study: 105 underwent single-site laparoscopic orchiopexy and 118 underwent conventional laparoscopic orchiopexy. During single-site laparoscopic orchiopexy, 3 ports were inserted within the umbilicus.

**Results:**

No differences were observed between the groups in terms of age and laterality. For unilateral undescended testes, the operating time was longer in the single site group than in the conventional group at the early stages (55.31 ± 12.04 min vs. 48.14 ± 14.39 min, *P* = 0.007), but it was similar to the conventional group at the later stages (48.82 ± 13.49 min vs. 48.14 ± 14.39 min, *P* = 0.78). Testicular ascent occurred in one patient from each group. There was no significant difference in the success rate between the single-site group and the conventional group (99.0% vs. 99.2%, *P* = 0.93). In the single-site group, no visible abdominal scarring was observed, while in the conventional group, there were two noticeable scars on the abdomen.

**Conclusion:**

Single-site laparoscopic orchiopexy offers superior cosmetic results and comparable success rates compared to conventional laparoscopic orchiopexy for palpable undescended testes.

**Supplementary Information:**

The online version contains supplementary material available at 10.1007/s00383-023-05630-8.

## Introduction

Undescended testis (UDT) is one of the most common congenital malformations in male infants, with an incidence rate of 1% at 1 year of age [1, 2]. Among UDT cases, palpable testes are the most common. Conventional laparoscopic orchiopexy (LO) has been shown to be effective in treating palpable testis [3–5]. However, a notable drawback is the presence of visible incision scars on the abdomen.

In recent years, laparoendoscopic single-site surgery (LESS) has been increasingly used in the field of urology [6]. In 2011, single-site LO was introduced in the pediatric population, offering the advantage of a concealed incision, which was well-received by parents [7]. However, existing reports are limited and primarily focus on non-palpable testes [8, 9].

There were few reports on the efficacy of single-site LO for palpable testes and many of these works included a small number of participants [10]. Herein, we conducted a prospective cohort study with a large sample size, to investigate whether single-site LO can achieve favorable success rates and better cosmetic results than conventional LO for palpable UDT.

## Materials and methods

### Patients

We prospectively studied patients with UDT between July 2021 and June 2022. Patients with palpable UDT and those with affected testes located in the inguinal canal were included. The exclusion criteria were as follows: (1) presence of disorders of sex development (DSD) or chromosomal anomalies; (2) history of inguinal surgery; (3) history of hormonal therapy.

Between July 2021 and June 2022, 261 patients with UDT were admitted to our ward. Twenty-nine patients with non-palpable testes and three patients with a history of inguinal surgery were excluded. Six patients with DSD or chromosomal anomalies were also excluded. In total, 223 patients were included in our study, and the surgical procedure was performed at the parents’ request. There were 105 patients in the single-site LO group (single site group) and 118 patients in the conventional LO group (conventional group). Patients were followed up at the outpatient clinic after discharge. Follow-up visits were performed up to 12 months after surgery to assess the testicular position and umbilical wound by physical examination and ultrasonography.

The variables included age, operating time, complications (classified by the Clavien-Dindo system), hospital stay, postoperative testicular location, testicular volume (confirmed by ultrasonography), testicular atrophy, success rate, and cosmetic appearance. Testicular atrophy was defined as the presence of a nubbin or impalpable testis at the follow-up visit, confirmed by Doppler ultrasonography. The success rate was defined as a proper testicular position without testicular atrophy or ascent. The 12-month period was divided into the early stage (first 6 months) and late stage (last 6 months). Testicular hypotrophy was defined as a reduction > 50% of the testicular volume compared to the contralateral, normally descended testis [11].

### Surgical procedure

**Fig. 1:**
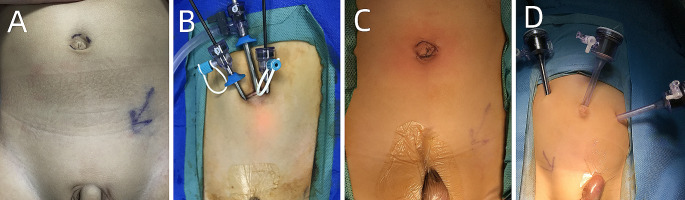
**A.** Location of the three incisions in the umbilicus for left undescended testis in single-site laparoscopic orchiopexy **B.** A 5-mm port for the camera was placed in the middle of umbilicus, and two 3-mm ports for instruments were located on each side **C. **Cosmetic appearance after accomplishment of single-site laparoscopic orchiopexy **D.** Location of three ports in conventional laparoscopic orchiopexy

**Fig. 2:**
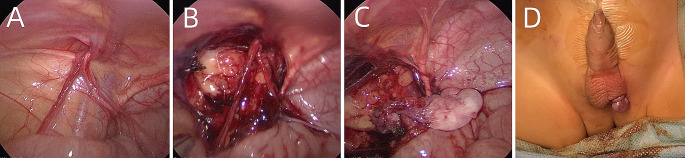
Surgical procedure of single-site laparoscopic orchiopexy for left undescended testis **A.** Preoperative findings **B.** Peritoneum over the testicular vessel and vas deferens was incised and dissected **C.** The testis was taken to the abdominal cavity and the gubernaculum was transsectioned **D.** The testis was delivered into the subdartos pouch from the abdominal cavity

During single-site LO, the patients were positioned supine with a 30° elevation on the affected side. Three small incisions were made along the border of the umbilicus (Fig. 1A). A 5-mm port was inserted for the camera through the central incision. Two 3-mm ports for the instruments were placed on either side of the first port within the umbilicus (Fig. 1B, C). The port on the affected side was positioned relatively higher, while that on the contralateral side was placed lower, resulting in a triangular configuration within the umbilicus (Fig. 1A). The peritoneum covering the testicular vessel and vas deferens was incised and dissected at the internal inguinal ring level (Fig. 2A, B). Releasing the testicular vessels from the peritoneum and retroperitoneum increased the length of the testicular vessels. The testis was brought into the abdominal cavity, and the gubernaculum was transsectioned (Fig. 2C). An ipsilateral scrotal incision was made at the bottom of the scrotum, creating a subdartos pouch. Forceps were passed through the scrotal incision into the abdomen. The testis was grasped at the gubernaculum and delivered into the subdartos pouch (Fig. 2D). Additional dissection was performed if tension was present in the vessel. Typically, the testis was brought down through the medial side of the inferior epigastric vessels to the scrotum, thus, shortening its distance.

In conventional LO, the first port of the laparoscope was placed in the umbilicus. Two 5-mm ports were introduced on either side of the abdomen at the umbilicus level along the midclavicular line (Fig. 1D). The surgical procedure in the abdominal cavity was the same as that in single site LO.

### Institutional review board approval

This study was approved by the ethics committee of our hospital, and informed consent was obtained from the guardians of all patients.

### Statistical analysis

Continuous data were compared using a t-test or covariance analysis. Categorical variables were compared using the chi-squared test. All statistical analyses were performed with a two-sided significance level of 0.05, using SPSS software (version 26.0; IBM Corp., Armonk, NY, USA).

## Results

In the single-site group, 105 patients underwent single-site LO, including 89 with unilateral UDT and 16 with bilateral UDT. Their mean age was 2.42 years (range, 6 months–12 years). In the conventional group, 118 patients underwent conventional LO, including 98 unilateral UDT and 20 bilateral UDT cases. The mean age was 2.21 years (range, 7 months to 13 years) (Table 1).

**Table 1:** Demography and surgical outcomes of single-site laparoscopic orchiopexy versus conventional laparoscopic orchiopexy

Variable	Single-site group	Conventional group	P
Number of patients(testes)	105 (121 testes)	118 (138 testes)	
Age (range)	2.42 ± 2.82	2.21 ± 2.36	0.54
Unilateral/bilateral	89/16	98/20	0.73
Operating time (min)	55.84 ± 16.02	52.82 ± 17.69	0.19
Early stage operating time (min) unilateral	55.31 ± 12.04	48.14 ± 14.39	0.007
Late stage operating time (min) unilateral	48.82 ± 13.49	48.14 ± 14.39	0.78
Pre-operative testicular hypotrophy	26 (24.8%)	29 (24.6%)	0.97
Post-operative testicular hypotrophy	18 (17.1)	22 (18.6%)	0.77
Testicular volume growth (ml)	0.07 ± 0.11	0.06 ± 0.12	0.33
Complications	3 (2.9%)	4 (3.4%)	0.82
Hospital stay(days)	3.43 ± 0.97	3.31 ± 1.04	0.36
Testicular atrophy	0	0	> 0.05
Testicular ascent	1	1	0.93
Redo-orchiopexy	1	1	0.93
Success rate	99.0%	99.2%	0.93

There were no significant differences between the groups in terms of age, laterality, operating time, or hospital stay. In the early stages, the operating time was longer in the single-site group than in the conventional group for the unilateral procedure (55.31 ± 12.04 min vs. 48.14 ± 14.39 min, *P* = 0.007). Nevertheless, in the later stages, the operating time in the single site group was similar to that of the conventional group (48.82 ± 13.49 min vs. 48.14 ± 14.39 min, *P* = 0.78). In the single site group, there was one case that required conversion to conventional LO in a 7-month-old boy who was underweight due to manipulation difficulty in the limited volume of the abdominal cavity.

**Fig. 3:**
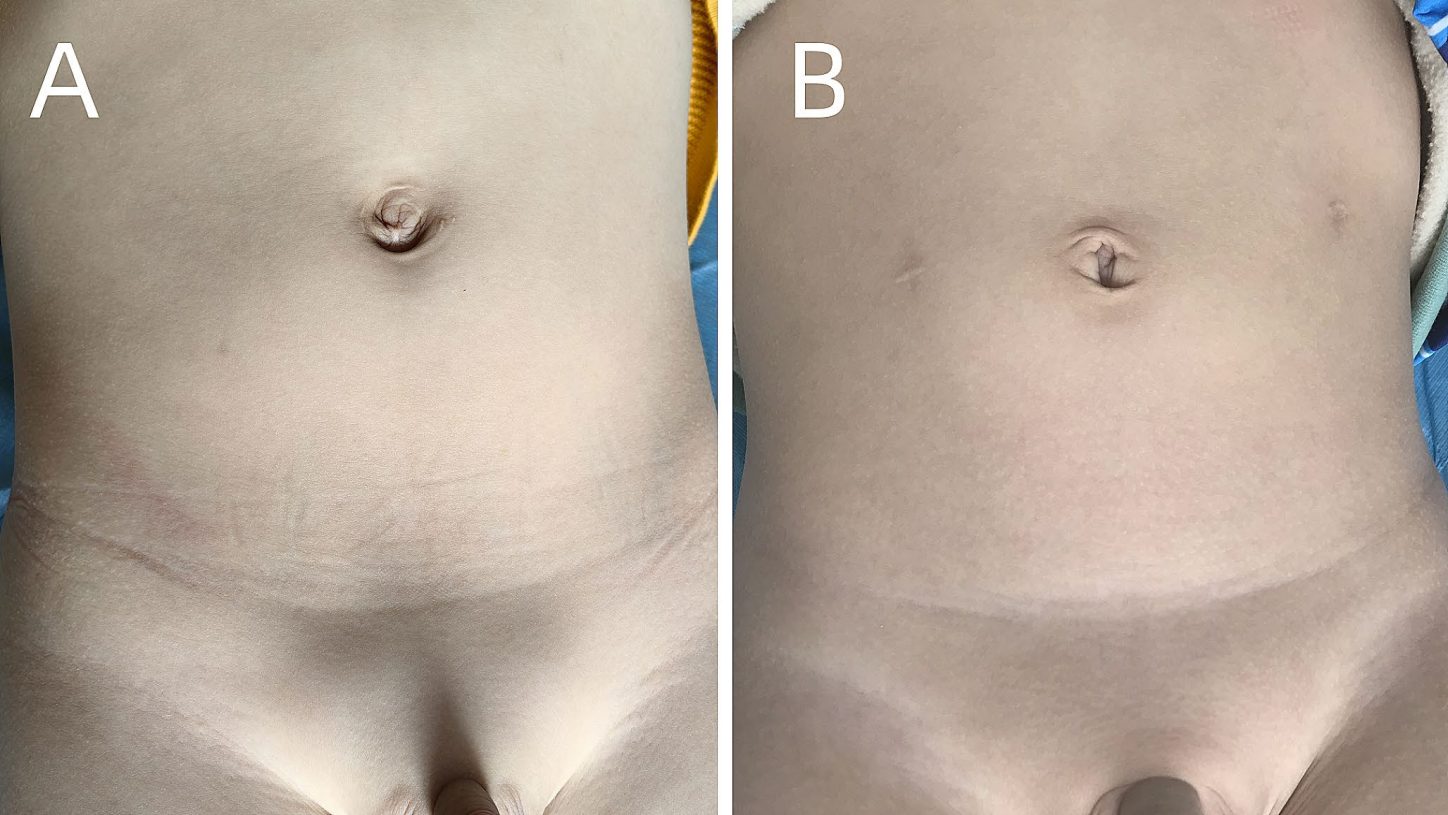
Cosmetic appearance after laparoscopic orchiopexy **A.** Cosmetic appearance after single-site laparoscopic orchiopexy. The incision scars were hidden in the umbilical fold **B.** Cosmetic result after conventional laparoscopic orchiopexy. Two noticeable incision scars were observed on the abdomen

After the 1-year follow-up, there was one patient in each group with testicular ascent which required reoperations. The remaining testes were located in the scrotum without testicular atrophy. There was no significant difference in the success rate between the single-site group and the conventional group (99.0% vs. 99.2%, *P* = 0.93). Ultrasonography revealed a testicular volume growth of 0.12 mL in the single-site group and 0.11 mL in the conventional group (*P* = 0.2). In the single-site group, there was no visible scar on the abdomen, and the umbilical scars were hidden (Fig. 3A). Their parents were satisfied with the cosmetic appearance. In the conventional group, there were two noticeable scars on the abdomen (Fig. 3B).

There were 3 complications in the single-site group (2.9%), including 2 hematomas (Grade I, Clavien-Dindo Classification), and 1 testicular ascent (Grade III). Similarly, there were 4 complications in the conventional group (3.4%), including 3 hematomas (Grade I), and 1 testicular ascent (Grade III). There was no significant difference in the complication rates between the two groups (*P* = 0.82).

## Discussion

To the best of our knowledge, this is the largest study evaluating single-site LO in comparison to conventional LO for palpable UDT in the pediatric population.

Our study revealed that single-site LO is a favorable technique for palpable UDT. It has a superior cosmetic appearance and a success rate comparable to that of conventional LO. Single-site LO is a safe, effective, and convenient technique that offers satisfactory cosmetic results. This method can be used extensively in children with palpable UDT.

Single-site LO has been increasingly used recently and offers several advantages. First, it provides favorable cosmetic outcomes. The small incision is hidden in the umbilicus, which results in no visible scar on the abdomen. The top and bottom of the umbilical ring are typically used as landmarks for the cranial and caudal ends of the skin incision to hide the incision in the umbilicus [12]. All parents were extremely satisfied with the cosmetic results. Other studies have also shown similar results [9, 10]. Wang et al. illustrated that a transumbilical single-site multiport procedure resulted in a smaller scar and less pain [13]. The incision is smaller than that used for the single-port procedure. Sultan et al. reported that single-port orchiopexy needs a 2.5-cm incision to accommodate a Triport (Advanced Surgical Concepts, Wicklow, Ireland) [7]. However, the 2.5-cm incision was longer than the 5-mm camera trocar and two 3-mm instrument ports. In our procedure, the incision was < 1.5 cm (Fig. 3A). Thus, the advantages of single-site surgery include superior esthetics with a smaller scar and less pain, and it is considered a less burdensome surgery for pediatric patients [14].

Second, single-site LO has a high success rate. In our study, only one patient had testicular ascent, and the procedure had a success rate of 99%. It can completely release the spermatic cord and simultaneously complete the diagnostic exploration and treatment. Thus, most patients have a scrotal testicular location without testicular atrophy. Both groups showed testicular volume growth after surgery, indicating that most testes grew well. The surgical results of single-site LO were comparable to those of conventional LO. Previous reports have also revealed that single-site LO had favorable results [9, 10]. A previous study revealed that laparoscopic single-site orchiopexy also has the potential to accomplish diagnosis and treatment with good cosmetic outcomes and less injury. Thus, single-site LO is a feasible and effective technique for the treatment of palpable inguinal UDT in children [10].

Third, single-site LO is convenient and easy to master. This technique uses common trocars and instruments without the need for special instruments. The use of adjacent puncture sites for instrumentation eliminates the need for commercial multichannel ports [15]. In selected patients, laparoendoscopic single-site surgery (LESS) for urological indications using conventional laparoscopic instruments has been shown to be safe and feasible, with no additional cost [16]. Although instrument crashing remains a concern in single-site surgery, our technique involved placing the two instrument ports 1 cm apart, effectively reducing the occurrence of crashing. As a result, the learning curve for this procedure is not extensive. In our experience, the initial operating time was 55 min in the single site group; however, it decreased to 48 min after 6 months, similar to that in the conventional group. This indicates that surgeons can quickly acquire proficiency in performing this procedure.

Limitations of single-site procedures include restricted motion and instrument crossings. Therefore, it is difficult to perform this procedure in a small abdominal cavity. LESS is accompanied by a lengthy learning curve and technical limitations associated with existing instrumentation. There is less range of motion, instrument crossing, and more instrument clashing during single-port surgery [7]. In our study, we encountered a situation where a trocar had to be added to the abdomen to complete orchiopexy for a 7-month-old underweight infant. In thin infants, this procedure should be performed with caution. Surgeons require training and experience in regular orchiopexy to perform single-site surgery proficiently. Therefore, further investigation is warranted to explore methods that can reduce the associated difficulties.

There are guidelines for single-site procedures. First, as single-site LO is known to be more challenging than standard LO, the locations of the three incisions need to be prospectively designed. The affected-side incision should be located higher than the contralateral instrument port, with the three incisions forming a triangle. This provides an acceptable distance between the two instruments (Fig. 1A, B). Second, for thin infants, preparation should be done for conversion to regular orchiopexy and the parents should be informed about the possible need for conversion before surgery.

There were differences between the results of our study and those of previous reports. Specifically, in previous works, patients had almost non-palpable testes [7–9]. However, our patients had palpable testes located in the inguinal canal; thus, surgeons needed to pull the testes back to the abdomen and transect the gubernaculum. Spermatic cords and testes were carefully pulled, as excessively strong traction would cause injury to the spermatic vessels. Therefore, we should be aware of the anatomical structure of the spermatic cord, dissect the peritoneum on the spermatic vessel, softly pull the cord, dilate the internal ring, and return the testes to the abdomen.

This was a prospective study that included a large number of cases, which was a strength of this study. Moreover, all testes were measured using ultrasonography before and after surgery, which provided objective and reliable data.

This study had some limitations. First, this was not a randomized, double-blind study. Second, long-term follow-up is required to confirm the superiority of this technique.

In conclusion, single-site LO offers superior cosmetic results and comparable success rates to conventional LO for palpable UDT. It seems to be an effective, safe, and convenient technique that achieves satisfactory cosmetic results. It can be used extensively for palpable UDT in the pediatric population.

## Electronic supplementary material

Below is the link to the electronic supplementary material.

Supplementary Material 1

## Abbreviations

UDT: undescended testis
LO: laparoscopic orchiopexy
LESS: laparoendoscopic single-site surgery
DSD: disorders of sex development
